# Happier People Live More Active Lives: Using Smartphones to Link Happiness and Physical Activity

**DOI:** 10.1371/journal.pone.0160589

**Published:** 2017-01-04

**Authors:** Neal Lathia, Gillian M. Sandstrom, Cecilia Mascolo, Peter J. Rentfrow

**Affiliations:** 1 Computer Laboratory, University of Cambridge, Cambridge, United Kingdom; 2 Department of Psychology, University of Cambridge, Cambridge, United Kingdom; University of Tennessee Health Science Center, UNITED STATES

## Abstract

Physical activity, both exercise and non-exercise, has far-reaching benefits to physical health. Although exercise has also been linked to psychological health (e.g., happiness), little research has examined physical activity more broadly, taking into account non-exercise activity as well as exercise. We examined the relationship between physical activity (measured broadly) and happiness using a smartphone application. This app has collected self-reports of happiness and physical activity from over ten thousand participants, while passively gathering information about physical activity from the accelerometers on users' phones. The findings reveal that individuals who are more physically active are happier. Further, individuals are happier in the moments when they are more physically active. These results emerged when assessing activity subjectively, via self-report, or objectively, via participants' smartphone accelerometers. Overall, this research suggests that not only exercise but also non-exercise physical activity is related to happiness. This research further demonstrates how smartphones can be used to collect large-scale data to examine psychological, behavioral, and health-related phenomena as they naturally occur in everyday life.

## Introduction

A sedentary lifestyle has been linked with many poor health outcomes. Time spent sitting is associated with increased risk of becoming obese, developing diabetes, cancer, and cardiovascular disease [1–3], and with increased risk of all-cause mortality [4]. Research suggests that—beyond exercise—small, cumulative, ‘non-exercise activity,’ such as standing and walking in the course of daily functioning, contributes to avoiding these negative outcomes and increasing general health [5]. A complementary question emerges: Are patterns of physical activity throughout the day also related to psychological health (e.g., happiness)?

To date, studies examining the relationship between happiness and physical activity have focused on exercise, finding mixed results. Some studies have found that happier people report exercising more [6, 7], while others have found no relationship between happiness and exercise [8, 9]. Much of this past research has relied solely on retrospective self-reports, on data collected at only one time period, and on small samples. Thus, no systematic research has examined links between happiness and behavioral markers of physical activity over time in a large, heterogeneous sample.

Every day, healthy individuals routinely engage in non-exercise physical activities. Because such activities are so common and, unlike exercise, do not require special planning or preparation, people may not be aware of the frequency or extent to which they stand, walk, or fidget throughout the day. As a result, assessing non-exercise activity using only self-reports risks generating coarse and inaccurate estimates. However, with the proliferation of smartphones that contain accelerometers—sensors that detect the phone's movement—it is possible to obtain objective estimates of physical activity throughout the day.

Ecological momentary assessment (EMA) is a powerful methodology for collecting in situ and in vivo momentary information about individuals’ psychological states, activities, and social contexts as they occur in everyday life [10]. Over time, such information provides rich (in the sense of quality) and dense (in the sense of quantity) information about individuals’ naturally-occurring daily lives. Smartphones provide a useful platform for EMAs because people carry their phones with them most of the time, so they can complete momentary assessments anywhere and (almost) any time. Moreover, smartphones are equipped with an array of built-in sensors capable of collecting relevant data unobtrusively at very high resolution. For example, the accelerometer detects the phone’s movement and can serve as a valid indicator of physical activity. Thus, with the combination of momentary self-report assessments and smartphone-sensor technology, smartphone-based EMAs offer a powerful platform for collecting data that, until recently, has been inaccessible to social scientists [11].

A number of studies have used EMAs to examine daily fluctuations in happiness. Results from these studies indicate, for example, that time spent with others is associated with increases in happiness [12]. However, all previous work in this area has relied on self-reports of both happiness and behavior. With respect to physical activity, most previous studies that have investigated a link with happiness have relied on self-reports of physical activity [8, 13, 14]. One study has measured physical activity objectively, using a physical accelerometer device attached to the hip [15]. However, the use of a physical device makes it challenging to recruit a large number of participants. In contrast, smartphone-based EMAs offer a valuable tool for investigating the link between happiness and physical activity in daily life from the large number of people who carry a smartphone, and with much more granularity than is possible with traditional, self-report methods.

The aim of the present investigation was to examine the link between total physical activity (including non-exercise physical activity) and happiness. We used longitudinal data from over 10,000 individuals who downloaded a mood-tracking smartphone application we developed. The app uses experience sampling (i.e., a form of EMA) to collect self-reports of happiness and physical activity, while at the same time passively collecting behavioral data from the accelerometer in the smartphone [16]. Several studies have demonstrated how the accelerometer can be used to detect the activities, postures, and movements of smartphone users [17, 18]. The integration of experience sampling and mobile sensing technology provides a powerful platform for collecting objective and longitudinal data at a large scale.

## Materials and Methods

### Participants

Participants were members of the general public who downloaded the freely-available app, described below, from the Google Play store and installed it on their Android phone. The analyses reported herein include all users who provided data on all four happiness measures (described below) from February 2013, when the app was released, to June 2014, when we began the analyses. A total of 12,838 users completed at least one self-report survey; 10,889 of them provided demographic information. Of these, 43% were female, 54% were male, and 3% did not provide information about their gender. Users indicated their age by selecting a 10-year range. The mode was 25–34 years of age (N = 4,098; 38%), but large numbers of users were either just younger (15–24; N = 1,896; 17%) or just older (35–44; N = 3,426; 31%). A total of 85 users (1%) did not provide information about their age. More than half of the sample was White (N = 7,382; 68%). The next most represented ethnicity was Asian (N = 1,386; 13%), and 526 users (5%) did not provide ethnicity information.

Each analysis that follows refers to a different subset of users (e.g., those who provided information about some combination of happiness, self-reported physical activity and/or sensed physical activity). We thus provide further information in the Results section about the exact subset of users for each analysis.

### The Mood-Tracking Application

The app was designed to study happiness and behavior. It collects self-report data through surveys presented on the phone via experience sampling. By default, the app sends two notifications at random moments of the day between 8AM and 10PM, at least 120 minutes apart from one another. Clicking on a notification launches a momentary assessment, which includes measures of current affect (i.e., mood), and measures assessing a single aspect of current behavior or context (e.g., physical activity, location, social interactions). In addition to the notification-driven surveys, the app also allows for self-initiated surveys. These included longer measures of affect, and measures assessing multiple aspects of behavior and context. (Please see the S1 File for a complete list of self-report measures.)

As well as collecting self-report data, the app also uses open-sourced software libraries [19] to periodically collect behavioral data from sensors in the phone (e.g., accelerometer). The data collected through the app are stored on the device's file system and then uploaded to a server when the phone is connected to a Wi-Fi hotspot.

The app was designed to be a tool to facilitate self-insight, providing feedback about how participants' affect relates to context and activity. When first installing the app, participants could only access feedback related to how their affect varies by time of day (e.g., morning, afternoon, evening, night). In an effort to maintain user engagement over a period of weeks, participants could receive additional feedback by ‘unlocking’ additional feedback screens, each of which had a particular theme (e.g., physical activity, location, social interactions) that determined which behavior and context questions were asked in the self-report surveys. Each successive feedback screen became available to be unlocked after a one week interval, and was unlocked by completing a measure of life satisfaction. The fourth screen that was available to be unlocked provided feedback related to physical activity. (Please see the S1 File for a complete list of self-report measures.)

This application was reviewed by and received approval from the ethics board in the Computer Laboratory at the University of Cambridge prior to release.

### Measures

#### Happiness

**Fig 1:**
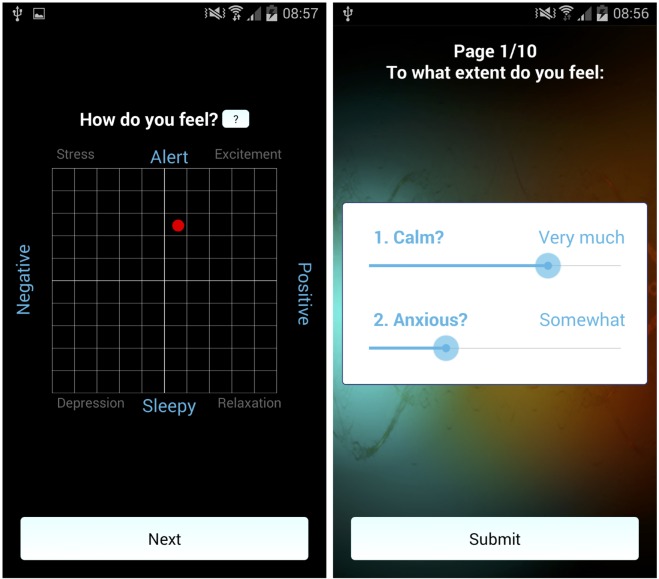
Self-Reported Mood in the app. From left to right: (a) Measuring happiness in the app using the affect grid: users select a point on a grid that quantifies valence (horizontally) and arousal (vertically). (b) Measuring happiness with PA/NA adjectives.

The app allowed users to track and quantify their happiness in various ways. On each self-report survey, whether notification-driven or self-initiated, users indicate their current feelings by tapping on a two-dimensional affect grid (see Fig 1A), where the x-axis denotes valence, from negative to positive, and the y-axis denotes arousal, from sleepy to alert [20].

Users also rate their current positive affect (PA) and negative affect (NA) by indicating the extent to which various adjectives describe their current mood, using a 7-point sliding scale, with end-points at 1 (*Not at all*) and 7 (*Extremely*; see Fig 1B). The mood adjectives, primarily drawn from the PANAS-X [21], correspond to each grid quadrant: high arousal/negative valence (angry, hostile, afraid, anxious, jittery), high arousal/positive valence (attentive, interested, alert, excited, enthusiastic), low arousal/positive valence (calm, relaxed, content), low arousal/negative valence (sad, lonely, depressed). On notification-driven surveys users rated two adjectives (see S1 File for more details), whereas, on user-initiated surveys, they rated eight adjectives (two from each grid quadrant: anxious, angry; alert, enthusiastic; calm, relaxed; sad, lonely).

Finally, users also complete a broader measure of life satisfaction (SWLS, [22]) each time they unlock a stage of the app. Users indicate the extent to which they agree with each of 5 statements using a 7-point scale, with end-points at 1 (strongly disagree) and 7 (strongly agree). These were the only measures of happiness in the app.

For each user who responded to each of these measures (grid, PA, NA, SWLS) at least once, we created a happiness composite score. Following Diener and Seligman [9], we computed a z-score for (i.e., normalized) each of the four measures, which were moderately correlated with each other, 0.24 <|r'|s < 0.57. Then we added together the z-scores for the user’s average grid valence, PA, NA (reverse-scored), and SWLS. Users provided a median of 29 affect grid ratings (min = 2, max = 1369), 34 mood adjective ratings (min = 1, max = 1202), and 2 satisfaction with life ratings (min = 1, max = 23).

We validated this composite happiness score by examining its relationship with self-reports of users' average amount of laughing and crying, which could be reported in a separate part of the application (i.e., a between-subject analysis). Happiness correlated positively with laughing, *r*(9,164) = .21, *p* < .001, *d* = .43, and negatively with crying, *r*(9,164) = -.18, *p* < .001, *d* = .38.

#### Physical Activity

**Fig 2:**
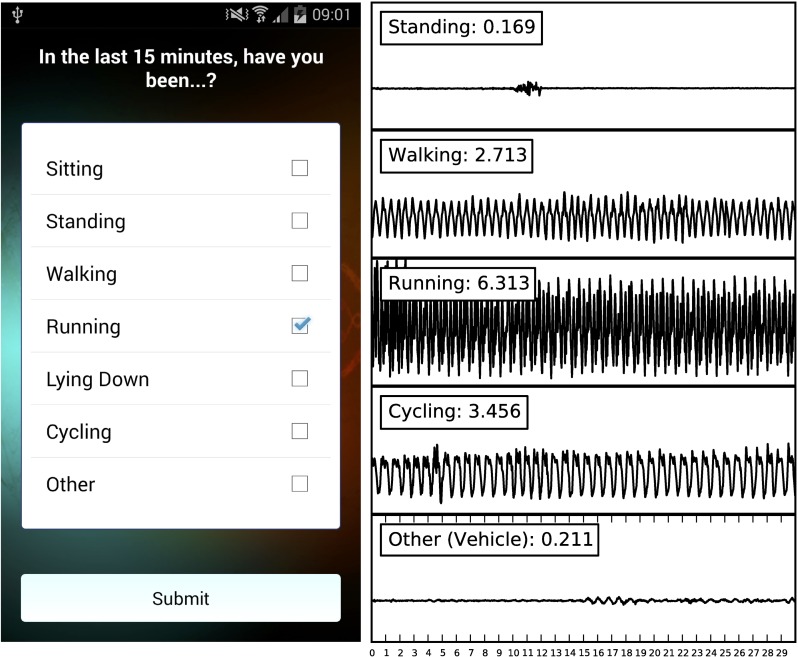
Physical Activity Data. From left to right: (a) How users self-report their recent physical activity, and (b) the magnitude of 30-second accelerometer samples collected on one device while performing each activity. The label (e.g., Walking: 2.713) contains the value of the feature computed from the given accelerometer sample; more physically demanding activities result in higher values.

One way the app assesses physical activity is through self-reports. Users indicate which activities they have been doing in the past 15 minutes (sitting, standing, walking, running, lying down, cycling or other; see Fig 2A). For each user we sum the number of active (walking, running, cycling) responses and divide this by the total number of responses (excluding ‘other,’ as it cannot be classified as either active or inactive), yielding an activity score that ranges from 0 to 1. For example, if a user reports walking, sitting, and standing in one 15-minute window, their activity score would be 0.33. Users reported sitting 8,127 times, standing 5,488 times, walking 5,399 times, running 1,076 times, lying down 5,708 times, cycling 533 times, and doing other activities 2,775 times. This is the only self-report measure of momentary physical activity in the app. (Please see the S1 File for a complete list of self-report measures.)

Physical activity is also sensed via the phone's accelerometer. For 15 minutes before each survey notification and at regular intervals throughout the day, the app measured the acceleration of the phone (see S1 File for details about the factors affecting frequency of the accelerometer measurements). An accelerometer captures the acceleration the device is subject to, in m/s^2^, in three dimensions (x, y, z). To score users' activity, we a) pre-processed the data, b) computed the axes' magnitude of acceleration (x^2^ + y^2^ + z^2^; see Fig 2B), which is often used in activity detection [23], and c) quantified activity using the standard deviation of this signal (see S1 File for details on this method and why we chose this particular measure).

## Results

### Validating accelerometers as a measure of physical activity

We measured the extent to which the smartphone accelerometer data aligned with self-reported physical activity. This analysis was run on the full set of self-reports of physical activity (i.e., each individual self-report for each user) that had corresponding accelerometer samples (i.e., samples at the same point in time as the self-reports; N = 23,419). A Pearson correlation found that the self-reported physical activity, referring to activity in the past 15 minutes, correlated with the activity score derived from the data sensed in the 15 minutes prior to the self-report, r(23,417) = .37, p < .001, d = .80. (We converted the correlation to a standardized mean difference with the formula d = 2r / sqrt(1-r2); see S1 File for analyses adjusting for possible lack of independence) This suggests that activity scores derived from smartphone accelerometers provided a reliable measure of physical activity, and therefore can be used as a coarse measure of physical activity in the absence of self-reports.

### Are people who are more physically active also happier?

We normalized the happiness scores (see Measures section for details) across the set of users who had rated their happiness on all four of the measures contributing to the happiness composite score, and had provided measures of their average physical activity, either self-reported (N = 9,130), sensed (N = 10,371), or both (N = 8,737). We then correlated the normalized happiness scores with the average of the person's physical activity (i.e., a between-subjects analysis). The results indicated that self-reported physical activity was positively related to happiness, r(9,128) = .08, p < .001, d = .16, as was sensed physical activity (as measured by the accelerometer), r(10,370) = .03, p = .002, d = .06 (see S1 File for results on individual happiness measures and results that control for personality). A regression predicting average happiness from both average self-reported and average sensed physical activity, entered as simultaneous predictors, found that both self-reported, β = .23, t(8,735) = 7.19, p < .001, and sensed physical activity, β = .06, t(8,735) = 2.00, p = .05, each independently predicted happiness. This supports the idea that objective measures can provide additional, unique insight into daily physical activity that goes beyond what we can learn from self-report measures alone. Taken together, these results suggest that happier people engage in slightly more physical activity (including non-exercise activity) than less happy people.

### Diurnal patterns of activity

Next, we turned from examining a person's overall average physical activity to examining a person's average hourly behavior. For each user, we created two profiles of accelerometer data: one representing average weekdays and one representing average weekend days. Each profile is a vector with 24 entries, where each entry contains the average of activity scores captured in that hour of the day. We then used the k-means++ clustering algorithm [24, 25] to create three groups of users who exhibited similar diurnal profiles of activity (see the S1 File for details on why we used k = 3).

**Fig 3:**
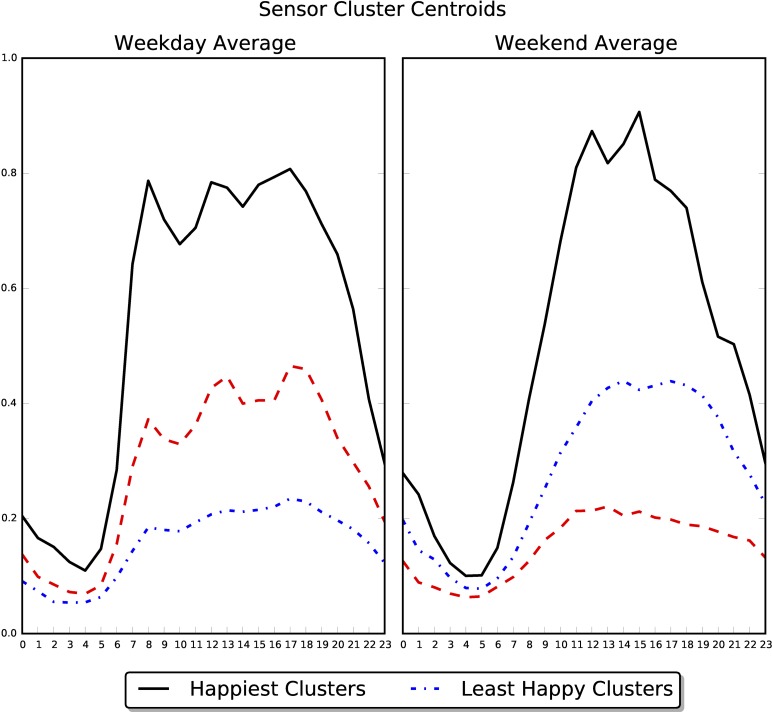
Centroids for the clusters generated from (left) weekday and (right) weekend activity profiles.

**Fig 4:**
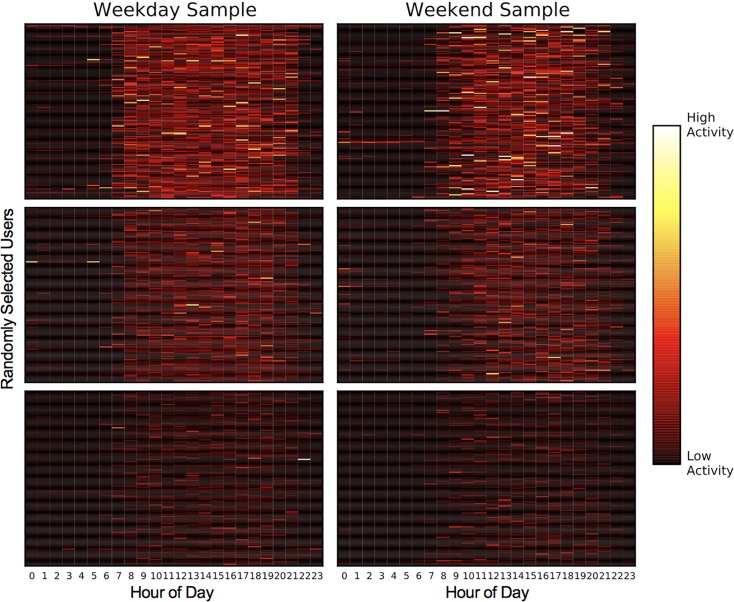
A random sample of 150 users from each of the weekday and weekend clusters: users in the active clusters were, on aggregate, happier than those in the less active clusters.

Although we did not specify any criteria or thresholds for the groups, the k-means method identified groups of users who exhibited high, medium, and low levels of diurnal activity; each group’s average activity is shown in Fig 3. Fig 4 visualizes the result by displaying the daily patterns of a random sample of 150 users drawn from each cluster. One-way ANOVA's, conducted separately on the weekday and weekend data, revealed that the clusters differed in happiness, both on weekdays, F(2, 10,294) = 40.22, p < .001, and on weekends, F(2, 9,633) = 33.74, p < .001 (results on individual happiness measures are reported in the S1 File). Post-hoc Tukey's tests revealed that on weekdays, people in the high (*M* = .25, *SD* = 2.86) and medium (*M* = .26, *SD* = 2.87) physical activity clusters were happier than people in the low activity cluster (*M* = -.27, *SD* = 3.12), *p*'s < .001, though the highly and moderately active groups did not differ from each other. On weekends, people in the high physical activity cluster were happier (*M* = .57, *SD* = 2.81) than people in the medium physical activity cluster (*M* = .23, *SD* = 2.87), *p* = .002, who were happier than people in the low physical activity cluster (*M* = -.15, *SD* = 3.04), *p* < .001.

Moreover, inspection of the clusters suggests that happy participants start their days earlier in the morning, end their days later in the evening, and display higher levels of physical activity throughout the day compared to less happy users. The levels of physical activity observed, however, were not intense or vigorous: Each cluster's average activity was smaller than the activity score that we manually collected while walking in a controlled setting.

### Are people happier in the moments when they are more active?

Finally, instead of looking at average behavior, we took advantage of the repeated measurements we had for each person by running multilevel models, with momentary happiness and physical activity measurements nested within person (i.e., a within-subjects analysis). (NOTE: We couldn’t use the happiness composite as reported in the previous analyses because the life satisfaction measure was not reported on a momentary basis.) We did these analyses using the lmer package in R [26]. We ran separate models predicting each of the various happiness ratings (affect grid; high and low arousal, positive and negative emotions from the mood adjectives) from the z-scored self-reported physical activity, the z-scored sensed physical activity, or both. Given that multilevel models essentially require a minimum of 3 data points per person, these analyses were performed on the subset of users who provided at least 3 measures of both self-reported and sensed physical activity (N = 2,005).

Self-reported physical activity predicted more positive valence on the grid responses, more intense high arousal positive affect, and less intense low arousal negative affect. Similarly, when sensed physical activity was used as a predictor in a multilevel model, it predicted more positive valence on the grid responses, more intense high arousal positive affect, and less intense low arousal negative affect, (see Table 1; see the S1 File for results that control for personality).

**Table 1:** Multi-level modelling results predicting affect from physical activity. Degrees of freedom are 2,005 for grid valence, 1,996 for high arousal positive affect, 1,975 for low arousal positive affect, 1,958 for high arousal negative affect and 1,958 for low arousal negative affect.

	Entered Individually		Entered Simultaneously	
Measure	Self-reported physical activity	Sensed physical activity	Self-reported physical activity	Sensed physical activity
Grid valence	β = .04, *t* = 7.31***	β = .03, *t* = 6.06***	β = .03***, *t* = 5.29	β = .02***, *t* = 3.71
Positive Affect				
High Arousal	β = .09***, *t* = 13.17	β = .05***, *t* = 7.89	β = .08***, *t* = 10.92	β = .02***, *t* = 3.27
Low Arousal	β = -.02, *t* = -1.77	β = -.01, *t* = -.70	β = -.01, *t* = -1.54	β = -.003, *t* = -.31
Negative Affect				
High Arousal	β = -.002, *t* = -.27	β = -.002, *t* = -.22	β = -.002, *t* = -.17	β = -.002, *t* = -.23
Low Arousal	β = -.04***, *t* = -4.81	β = -.03***, *t* = -3.81	β = -.03***, *t* = -3.63	β = -.02*, *t* = -2.19

* p < .05

*** p < .001.

Further supporting the idea that both objective measures and self-report measures independently predict happiness, analyses that examined both self-reported and sensed measures simultaneously found that both self-reported and sensed physical activity predicted more positive valence on the grid responses, more intense high arousal positive affect, and less intense low arousal negative affect (see Table 1).

## Discussion

Poor health has significant individual and societal costs. The current project showed that inactivity, which has been linked to poor physical health, is also linked to poor psychological health (i.e., lower happiness). Using a large-scale, public deployment of a mobile application that periodically assessed participants' happiness and passively measured physical activity, we discovered a modest but reliable association between happiness and physical activity. These findings have important implications for research on happiness, and also for behavioral science research methods.

It might be tempting to dismiss the results as spurious; when analyzing “big data”, such as the data used in this study, it is a common belief that *all* associations will be statistically significant. That is not our experience in working with this data set. Also, it is important to point out that the opposite is also true: when analyzing small data, relationships can appear to be statistically significant when there is in fact no association. In fact, with the smaller standard errors that come with “big data”, we can be more confident that there is, in fact, a relationship between two variables, though that relationship may be modest. The association with happiness reported in the current paper is modest in size, but reliably manifests both for self-reported (i.e., subjective) physical activity and for objectively sensed physical activity. Obviously there are many factors that contribute to happiness. Given that positive social relationships may be the single most important factor contributing to happiness [27], we anticipate that the social interactions that underlie those relationships would have a strong influence on momentary happiness. Indeed people do report more positive affect when they are in social situations [28]. Future work that measures and statistically controls for this and other factors affecting happiness might reveal an even stronger relationship between happiness and physical activity. For now, given the size of the association, readers may wish to exercise caution when interpreting the importance of the relationship between physical activity and happiness.

The current findings extend previous research on the link between happiness and physical activity by demonstrating that regular physical activity—including non-exercise physical activity like standing, walking, and fidgeting—has a positive connection to psychological health. Experimental studies suggest that physical activity increases happiness [29, 30], but it is also possible that feeling happier leads to more activity. Although the design of the current study does not allow us to tease apart these competing hypotheses, the fact that our physical activity data were collected throughout the day suggests that happiness is linked to even non-exercise physical activity. Experimental investigations in which participants are instructed to increase or decrease their level of physical activity (both exercise and non-exercise) would shed light on the degree to which physical activity impacts happiness.

This study provides a compelling demonstration of how smartphones can collect large-scale data to examine psychological and behavioral phenomena as they occur in daily life [31, 32]. Indeed, mobile technology offered a methodology for investigating the link between physical activity and happiness that was more reliable and fine-grained than traditional methodologies. The results go beyond previous research using EMA for studying daily fluctuations in happiness by examining *objective* behavior. Nonetheless, it would be misleading to imply that smartphone-based EMAs can overcome all the limitations of traditional self-report and laboratory-based experiments. Indeed, there are broad and specific limitations associated with collecting data from smartphone sensors. Carefully controlled experiments are the cornerstone of science, and data collected in the wild are inherently messy and harder to interpret than data obtained from lab-based observations. Thus, smartphone-based studies allow for examining phenomena in the real world at the expense of losing control over all the events that naturally occur in life. More specific limitations are that even though smartphones are consistently proximal to their owners [33], there are likely to be instances where users are active without carrying their phones; the data in this study are likely to underestimate actual activity and to miss out altogether on rigorous exercise. Moreover, collecting continuous data from smartphones remains challenging, as this will quickly consume the device's battery. Smartphones are continuing to improve: more modern devices that have been released since our study include co-processors and dedicated physical activity tracking apps that are increasingly efficient at assessing diurnal physical activity. Future studies could make use of data from these apps (although some questions around accuracy remain [34]). Finally, it is also difficult to gauge the extent that receiving feedback from any app could alter both mood and behaviour [35]. Some of these limitations could be relieved by tracking daily activity with wearable sensors (see [36] for a review), but this approach remains, for now, expensive and impractical for assessing activity at the large scale necessary to detect the effects of small changes in behavior.

## Conclusion

The frequency with which people physically move throughout the day, even if that movement is not rigorous exercise, is associated with both physical health and happiness. The current research reveals the important connection between physical and psychological processes, indicating that even slight changes in one has consequences for the other. The present findings validate the use of the smartphones to passively measure the daily activities of a diverse cohort of people, and pave the way for future research concerned with links between psychological and behavioral processes.

## Supporting Information

S1 File (DOCX)
