# Supplementary material for: Happier People Live More Active Lives: Using Smartphones to Link Happiness and Physical Activity
Source: PLoS One. 2017 Jan 4;12(1):e0160589. doi: 10.1371/journal.pone.0160589 (PMC5213770; doi:10.1371/journal.pone.0160589)
Supplement: S1 File — (DOCX) [file pone.0160589.s001.docx]

**Supporting Information**

**Materials and Methods**

**Participants**

Our mood tracking application was released to the public as a free download on the Google Play store in February 2013. A press release from Cambridge University in May 2013 and the subsequent coverage from a variety of newspapers and social media mentions helped to disseminate the app internationally.

Because the app was running only on Android-powered phones, all participants were Android users. The demographic characteristics of the participants in our sample are comparable to the demographic characteristics of Android users [37]. Given that Android represents more than half of the smartphone markets in North America, Europe, and parts of Asia, there is little reason to suspect that our sample is not representative of the majority of smartphone users.

**The Mood Tracking Application**

Upon first opening the app, participants were presented with a consent form explaining the nature of the study and the data collected. The consent form assured participants that “any data this app collects will remain private and be securely stored'” and that “only members of the research team will be able to access your data.” Participants were also told they could request a copy of their data and have all or any part of their data deleted. Once participants provided consent, all the features of the app were activated and participants were prompted to read a short tutorial of how to use the app. When a user logged-out or uninstalled the app, all the features of the app were disabled.

In addition to the notification-driven and self-initiated surveys, the app also contained profile surveys. The app's “Profile” section displayed a set of surveys that participants could voluntarily complete. The profile surveys included demographic questions, as well as assessments of psychological constructs including personality, social connectedness, gratitude, and personal values, which users could complete once. None of the psychological assessments from the profile surveys were included in the present analyses.

As described in the main text, users could unlock a new feedback screen after each week of using the app. As expected, the number of users who unlocked successive feedback screens dropped. Although 10,818 users provided at least one self-report, 10,135 users provided self-reports at feedback screen 1 (time of day), 5,165 users provided self-reports at feedback screen 2 (location), 3,839 users provided self-reports at feedback screen 3 (SMS patterns), and 2,913 users provided self-reports at feedback screen 4 (physical activity). However, given that users also reported on physical activity during the self-initiated surveys regardless of what feedback screen they had unlocked, a total of 9,130 users provided self-report information about their physical activity.

**Measures**

**Happiness**

On notification-driven surveys, users rated their current affect on the grid, and then rated two mood adjectives. One of the mood adjectives was chosen from the same quadrant as the response on the grid. For example, if the user reported that their mood was negative in valence and high in arousal, then they were asked to rate one of the following adjectives, chosen at random: angry, hostile, afraid, anxious, or jittery. The second adjective was chosen at random from the list of adjectives for the remaining 3 grid quadrants.

**Physical Activity**

We pre-processed the accelerometer samples as follows. Some accelerometer samples returned little or no data. This could be due to (a) faulty sensors, or (b) some Android devices not generating accelerometer events when the screen is turned off. We first removed any samples that had no values in any or all of the three axes. We also removed samples that had fewer than 100 values per axis, which was deemed insufficient for a 10-second sample. Additionally, some accelerometer samples contained values that were impossibly high (e.g., acceleration values greater than 1,000); we removed samples containing feature values that were above 20, a value that is already substantially higher that what would be experienced by a typical smartphone.

The average time between users' first and last accelerometer sample is 51.9 days. The accelerometer sampling in the app produces an average of approximately 20 minutes of sensor data per user, per day, uniformly at random. The app is set to collect sensor samples prior to creating a momentary survey notification and at regular 15-minute intervals throughout the day, but there are a variety of factors that further affect how much sensor data we captured from a user: users can manually increase/decrease the number of notifications they receive, some devices disable sensors when the phone is not in use, and some (although, we expect, few) users may manually disable the app's background sensing task.

We computed a large set of accelerometer features by extracting the mean, standard deviation and variance for each axis, and then using the same summary statistic to aggregate across axes, and then across a user's samples. We tested all of the extracted features by correlating them with self-reported physical activity. We chose to analyse the standard deviation of the magnitude since, compared to all the features we tested, it correlated most strongly with self-reported momentary physical activity.

**Other self-report measures**

Full details of all measures are available on request from the second author.

**Notification-driven momentary assessments**

Every momentary assessment included the affect grid, to assess mood, as well as questions that were tied to the stage the user was at. In Stage 1 (Time), in addition to the affect grid question, users also answered two questions about how their day was going. In Stage 2 (Location), users answered one question about where they were. In Stage 3 (SMS), users answered two questions about how active their social life had been. Stage 4 (Accelerometer) is the stage reported in the main text, in which users answered two questions about their physical activity. Questions in the remaining stages were only seen after completing Stage 4, so they would not have impacted the data reported in the current manuscript. The questions in these stages are as follows: stage 5 (Screen; two questions about device use), stage 6 (Microphone; one question about verbalizations, such as talking and laughing, and one question about noise in the environment), stage 7 (Calls; two questions about how active their social life had been), stage 8 (Personality; 5 questions about Openness, Conscientiousness, Extraversion. Agreeableness, Neuroticism), stage 9 (Sociability; 4 questions about recent social interations), stage 10 (Connectedness; one question) and stage 11 (Engagement; 5 questions about feelings of stress, challenge, creativity, interest, and enjoyment).

**Self-initiated surveys**

These surveys included questions about mood, as described in the happiness measures section of the main text. They also included all the questions from stages 1 through 7, as described above.

**Profile surveys**

These surveys were (generally) completed by users only once, and were not required. If a user had incomplete profile surveys, some text appeared on the app’s home screen prompting them to complete the next available profile survey. In addition to the demographic measures and the life satisfaction measure, which are reported in the main text, users could complete measures of: personality (10 questions), gratitude (6 questions), health (8 questions), sociability (8 questions), job satisfaction (5 questions), life values (22 questions), and connectedness (10 questions).

**Results**

**Happiness**

We note that the users in this study were not especially happy; they scored lower on the SWLS (*M* = 19.40, *SD* = 6.54) than users who responded to an online survey hosted by the British Broadcasting Corporation (*N* = 588,014; *M* = 23.80, *SD* = 7.01 [38]). They also scored lower than users who completed psychological tests on the MyPersonality Facebook application (*N* = 101,068; *M* = 21.90, *SD* = 6.85; [39]).

There were minor differences in happiness between demographic groups in this study. For example, male users were happier than the female users, *F*(1, 8,198) = 43.71, *p* < .001. However, this difference was only apparent in the grid valence responses, *F*(1, 8,198) = 97.4, *p* < .001, and PA ratings *F*(1, 8,198) = 131.7, *p* < .001, but not in the NA ratings *F*(1, 8,198) = 0.002, *p* > .25 or SWLS *F*(1, 8,198) = .81, *p* > .25.

Further, for those age ranges that had more than 50 people (i.e., excluding people over the age of 70), a one-way ANOVA found that age predicted happiness, F(6, 8,395) = 7.32, *p* < .001. With the exception of the youngest users (less than 14 years old), mean happiness increased with age from age 15 through 64, though post-hoc Tukey comparisons revealed few significant differences between age groups.

**Physical Activity**

Overall, users did not report being highly active; the most frequent activities were sitting (43%), standing (21%), walking (21%), and lying down (14%), while both running and cycling each appear in less than 1% of reports, which broadly agrees with other studies on self-reported physical activity [31]. In 40% of the 555,436 activity responses received, users reported having been engaged in more than one activity. The most frequent pairs were combinations of the most frequent activities (sitting and standing, sitting and walking, standing and walking; for example, 43.2% of reports that included walking also included sitting).

Our app collected 109,306,542 accelerometer samples during the time under investigation. Much like the self-reports, an overwhelming majority of these samples indicate very low amounts of activity. Approximately 30% of samples have activity scores below 0.03, whereas when one of the authors manually collected walking data, the activity score was above 2.

Unlike the happiness scores, there were no age differences in self-reported physical activity, *F*(4, 7361) = .64, *p* > .25, but there were gender differences, *F*(1, 7,320) = 18.72, *p* < .001, with men reporting more physical activity (*M* = .15, *SD* = .17) than women (*M* = .14, *SD* = .15).

**Validating accelerometers as a measure of physical activity**

Although we have no reason to believe that the relationship between one self-report and one accelerometer sample is not independent from the relationship between another self-report and another accelerometer sample, even if both self-reports and both accelerometer samples come from the same user, for completeness we tested this relationship using multilevel modelling. We ran a model predicting self-reported physical activity from the sensed physical activity (level 1), grouped by user (level 2). The sensed physical activity is a significant predictor of self-reported physical activity, b = .16, *t(*3,906) = 61.07, *p* < .001.

The correlation between self-reported and sensed activity was similar in size for females, *r*(10,394) = .36, *p* < .001, *d* = .77, and males, *r*(11,373) = .39, *p* < .001, *d* = .86. These results held when tested with a multilevel model instead: b = .18, *t*(1559) = 39.22, *p* < .001 for females and b = .16, *t*(1888) = 45.52, *p* < .001 for males. Thus, the assumption that males may be more likely to carry their phones in their pockets while females may carry their phones in a bag does not seem to impact the validity of the sensor-detected physical activity data.

**Are people who are more physically active also happier?**

As we reported in the main text, using between-subjects analyses we found that self-reported physical activity was correlated with happiness (using the composite measure), r(9,128) = 0.08, p < .001. If, instead of using the happiness composite, we look at the individual happiness measures, we see consistent patterns (see Table 2).

**Table 2**. Correlations between individual happiness measures and both self-reported and sensed physical activity.

|  | Grid valence | Positive affect | Negative affect | Satisfaction with life |
| --- | --- | --- | --- | --- |
| Self-reported physical activity | *r*=0.08***, *d*=0.17 | *r*=0.06***, *d*=0.11 | *r*=-0.05***, *d*=-0.10 | *r*=0.06***, *d*=0.11 |
| Sensed physical activity | *r*=0.03***, *d*=0.06 | *r*=0.06***, *d*=0.11 | *r*=0.02**, *d*=0.05 | *r*=0.03**, *d*=0.06 |

Degrees of freedom are 9,128 for self-reported physical activity, and 10,369 for sensed physical activity

** p < .01, *** p < .001

This effect did not seem to depend on the type of self-report; we find similar correlations when we look at only notification-driven or only self-initiated reports of physical activity (see Table 3).

**Table 3**. Correlations between self-reported physical activity and self-reported affect, comparing notification-driven vs. user-initiated self-reports.

|  | Grid valence | High arousal PA | Low arousal PA | High arousal NA | Low arousal NA |
| --- | --- | --- | --- | --- | --- |
| Notification-  Driven | *r*=0.09***, *d*=0.18 | *r*=0.07***, *d*=0.14 | *r*=0.05**, *d*=0.10 | *r*=-0.05**, *d*=-0.10 | *r*=-0.09***, *d*=-0.18 |
| Self-  Initiated | *r*=0.08***, *d*=0.15 | *r*=0.10***, *d*=0.21 | *r*=0.03**, *d*=0.06 | *r*=-0.05***, *d*=-0.09 | *r*=-0.06***, *d*=-0.12 |

Degrees of freedom are 2,970 for notification-driven self-reports, and 8,986 for self-

initiated self-reports

** p < .01, *** p < .001

Users could optionally provide self-reports of their personality. Of the 9,130 users who provided self-reports of physical activity, 890 also provided self-reports of personality. The relationship between average self-reported physical activity and average self-reported happiness (i.e., a between-subjects analysis), which was significant for the larger set of users, was marginal for the subset of users who provided personality data, *r*(888) = .06, *p* = .07, *d* = .12 (or, alternatively, for ease of comparison with the subsequent results, 𝛃 = 1.34, *t*(888) = 1.82, *p* = .07). Importantly, the size of this relationship was similar after controlling for personality; when average happiness was predicted from average self-reported activity and all of the big-five personality traits (Openness, Conscientiousness, Extraversion, Agreeableness and Neuroticism), activity remained a marginally significant predictor, 𝛃 = 1.02, *t*(888) = 1.67, *p* = .10. Each of the personality traits also predicted physical activity (𝛃 = .64, *t*(883) = 6.93, *p* < .001 for Extraversion).

Of the 10,371 users who provided sensed physical activity data, 946 also provided self-reports of personality. The relationship between average sensed physical activity and average self-reported happiness (i.e., a between-subjects analysis), which was significant for the larger set of users, was not significant for the subset of users who provided personality data, *r*(944) = .03, *p* = .38, *d* = .06 (or, alternatively, for ease of comparison with the subsequent results, 𝛃 = .79, *t*(944) = 0.88, *p* = .38). Importantly, the size of this relationship was somewhat larger after controlling for personality; when average happiness was predicted from average sensed activity and all of the big-five personality traits, activity remained a non-significant predictor, 𝛃 = .86, *t*(939) = 1.12, *p* = .27. All of the personality traits, except for agreeableness, also predicted physical activity (𝛃 = .59, *t*(939) = 6.51, *p* < .001 for Extraversion).

**Diurnal patterns of activity**

To analyse the effect of the k parameter on our clustering quality, we examined how the clusters produced from sensor data differed from one another with respect to self-reported physical activity, which was not taken into account when producing the clusters. We ran a series of one-way ANOVA's predicting self-reported physical activity from cluster membership. Post-hoc analyses using Tukey's test provided support for the choice of three clusters: When there are three clusters from the raw sensed data, they differ significantly from one another in self-reported momentary physical activity for both weekdays and weekends, *p*'s < .01. In contrast, when there are four or more clusters, at least one pair of clusters do not differ.

We note that the vectors we clustered are potentially sparse, if no data was sensed/reported during those hours. We quantified the sparsity of each profile by counting the number of missing values per user. The profiles based on self-reported data have missing values for over 78.6% of users on weekdays, and over 83.9% of users on weekend days (spiking to 90% of users during night hours, from 1AM to 6AM). For those hours that do have data, the average number of reports is slightly higher for weekdays (*M* = 1.73, *SD* = 0.29) than weekend days (*M* = 1.29, *SD* = 0.11). On the other hand, profiles based on sensor data are less sparse: There are missing values for 42.5% of users on weekdays and 52.8% of users on weekend days. Bins that did have data contained a much higher average amount compared to self-reports: Weekdays had *M* = 50.05 (*SD* = 28.8) and weekend days had *M* = 24.82 (*SD* = 14.3) minutes of accelerometer data.

In the main text, we reported that when users were clustered based on sensed physical activity, they differed in happiness, as measured by the happiness composite. The clusters are also significantly different in happiness when we look at individual happiness measures (see Table 4).

**Table 4**. One-way ANOVA’s predicting individual happiness measures from cluster membership.

|  | Grid valence | Positive affect | Negative affect | Satisfaction with life |
| --- | --- | --- | --- | --- |
| Weekdays | *F* = 63.91*** | *F* = 14.49*** | *F* = 14.29*** | *F* = 15.82*** |
| Week-ends | *F* = 48.34*** | *F* = 9.31*** | *F* = 18.75*** | *F* = 10.62*** |

Degrees of freedom are 2 and 10,294 for weekdays, and 2 and 9,633 for week-ends.

*** p < .001

In addition to examining clusters based on sensed physical activity, we also ran a series of one-way ANOVA's predicting happiness, normalized across the set of users with happiness scores, from membership of clusters generated with self-reported profiles. We found a similar result to the clusters produced with sensor data. On weekdays the clusters differed in happiness, *F*(2, 8,412) = 32.83, *p* < .001. Post-hoc Tukey's tests showed that the least happy cluster (*M* = -.10, *SD* = 3.05) was less active than the other two clusters, *p*'s < .001, which did not differ in happiness (*M* = .50, *SD* = 3, and *M* = .50, *SD* = 2.86). On weekends, the clusters also differed in happiness, *F*(2, 5,613) = 14.76,  *p* < .001. Post-hoc Tukey's tests showed that the least happy cluster (*M* = .04, *SD* = 2.99) was less active than the most happy cluster (*M* = .58, *SD* = 2.98), *p* < .001, but neither differed in happiness from the third cluster (*M* = .37, *SD* = 2.94).

**Are people happier in the moments when they are more active?**

Of the 2,005 users who provided self-reports of physical activity, 380 also provided self-reports of personality. The multi-level modelling results remain similar when personality traits are entered as simultaneous predictors (see Table 5). Personality traits also predict happiness; Neuroticism predicts all happiness measures, Extraversion predicts grid valence, high arousal positive affect and low arousal negative affect, and Conscientiousness predicts high arousal positive affect.

**Table 5**. Multi-level modelling results predicting affect from physical activity, controlling for personality.

|  | Entered Individually | | Entered Simultaneously | |
| --- | --- | --- | --- | --- |
| Measure | Self-reported physical activity | Sensed physical activity | Self-reported physical activity | Sensed physical activity |
| Grid valence | β = .05, *t* = 3.24** | β = .03, *t* = 2.10* | β = .04, *t* = 2.72** | β = .02, *t* = 1.17 |
| Positive Affect |  |  |  |  |
| High Arousal | β = .11, *t* = 7.17*** | β = .06, *t* = 4.01*** | β = .10, *t* = 6.28*** | β = .03, *t* = 2.04* |
| Low Arousal | β = -.03, *t* = -1.86 | β = -.02, *t* = -1.30 | β = -.03, *t* = -1.53 | β = -.01, *t* = -.92 |
| Negative Affect |  |  |  |  |
| High Arousal | β = .01, *t* = .59 | β = -.004, *t* = -.30 | β = .01, *t* = .70 | β = -.007, *t* = -.48 |
| Low Arousal | β = -.04, *t* = -2.52* | β = -.02, *t* = -1.50 | β = -.03, *t* = -2.19* | β = -.01, *t* = -0.78 |

Degrees of freedom are 380 for grid valence, 380 for high arousal positive affect, 378 for low arousal positive affect, 379 for high arousal negative affect and 377 for low arousal negative affect.

* *p* < .05, *** *p* < .001
